# Diagnosis of cytomegalovirus infection from clinical whole genome sequencing

**DOI:** 10.1038/s41598-020-67656-5

**Published:** 2020-07-03

**Authors:** Nanda Ramchandar, Yan Ding, Lauge Farnaes, David Dimmock, Charlotte Hobbs, Stephen F. Kingsmore, Matthew Bainbridge

**Affiliations:** Rady Children’s Institute for Genomic Medicine, 7910 Frost St Ste 240, San Diego, CA USA

**Keywords:** Clinical genetics, Microbial genetics, Paediatrics, Genetics, Medical research, Diseases, Infectious diseases

## Abstract

Rapid whole genome sequencing (rWGS) of peripheral blood has been used to detect microbial DNA in acute infections. Cytomegalovirus (CMV) is a herpesvirus capable of causing severe disease in neonates and immunocompromised patients. We identified CMV in patients undergoing diagnostic rWGS by matching reads that did not align to the human reference genome to a database of microbial genomes. rWGS was conducted on peripheral blood obtained from ill pediatric patients (age 1 day to 18 years). Reads not aligning to the human genome were analyzed using an in-house pipeline to identify DNA consistent with CMV infection. Of 669 patients who received rWGS from July 2016 through July 2019, we identified 28 patients (4.2%) with reads that aligned to the CMV reference genome. Six of these patients had clinical findings consistent with symptomatic CMV infection. Positive results were highly correlated (R^2^ > 0.99, *p* < 0.001) to a CMV-qPCR assay conducted on DNA isolated from whole blood samples. In acutely ill children receiving rWGS for diagnosis of genetic disease, we propose analysis of patient genetic data to identify CMV, which could impact treatment of up to 4% of children in the intensive care unit.

## Introduction

Rapid whole genome sequencing (rWGS) of peripheral blood can rapidly diagnose genetic diseases and impact clinical care within 24 h^1,2^. A recent study showed a diagnosis rate of 43% in critically ill patients with a resultant change in management in 33% of patients^1^. rWGS allows clinicians to make actionable diagnoses in critically ill patients who may have an underpinning genetic or metabolic disease and is increasingly becoming a part of standard care. The concept of applying rWGS to detect infectious pathogens has also been utilized^3–6^. While plasma polymerase chain reaction (PCR) is often used for detection of infectious organisms, we theorized that an advantage of sequencing whole blood is that organisms that reside intracellularly within white blood cells are included in rWGS reads. Cytomegalovirus (CMV) is human herpesvirus 5 (HHV-5) and capable of causing disease in critically ill patients^7^. It can be found both intracellularly and extracellularly in humans. CMV can cause hepatitis, marrow suppression (including thrombocytopenia), encephalitis, retinitis, and pneumonitis. In neonates, CMV may also manifest as growth restriction, microcephaly, neurologic defects (including sensorineural hearing loss), and intellectual disability. Prevalence of congenital CMV ranges from 0.2 to 2.0% of pregnancies. Although most infected infants will have only latent infection without any sequelae, up to 12%—representing approximately 9,600 children per year in the United States—will experience neurological sequelae related to congenital CMV infection^8^. Recent studies have demonstrated improved outcomes in neonates with congenitally acquired CMV infection if antiviral treatment is started within the first 30 days of life^9^. Identifying CMV infection in critically ill children who are undergoing rWGS has the potential to significantly influence their outcomes. We sought to retrospectively detect the presence of CMV in patients undergoing rWGS and to identify those in whom CMV may have contributed to the severity of their illness.

## Methods

Peripheral blood was obtained from acutely ill pediatric patients (age 1 day to 18 years) after parental consent and patient assent, where indicated, were obtained. Informed consent was obtained from all subjects or, if subjects were under 18 years, from a parent and/or legal guardian, including permission for publication. Research was conducted according to the principles of the Declaration of Helsinki and as approved by the University of California San Diego Institutional Review Board. All experimental protocols were approved by the University of California San Diego Institutional Review Board. Genomic DNA was extracted, paired-end genomic libraries were prepared without PCR, and sequenced (2 × 100 bp) on Illumina (San Diego, CA, USA) HiSeq 2,500, HiSeq 4,000 or NovaSeq 6,000 instruments, as previously described, to a whole genome redundant depth of ~ 40-fold^1^. Reads were aligned to the human genome (hg19) as previously described^1^. Unaligned reads were analyzed using an in-house built pipeline. Reads were first extracted from the whole genome binary alignment map (BAM) and realigned to the human genome with Burrows-Wheeler Aligner-Maximal Exact Match (BWA-MEM) using less stringent parameters (− c 10,000) to allow repetitive reads derived from the human genome to be aligned^10^. Reads with low quality (> 1/8th of the read length with a phred-like score of 1) or low complexity (> 25% of the read has the same dinucleotide) were removed. The remaining reads were aligned to a database of viral and bacterial genomes retrieved from the Microbial Genome Database (https://mbgd.genome.ad.jp/, accessed May 2017) and National Center for Biotechnology Information (https://www.ncbi.nlm.nih.gov/genome/viruses/) (accessed Apr 2017)^11^. Duplicate alignments were then marked and removed using Sambamba (https://github.com/biod/sambamba)^12^. Only reads of at least 100 consecutive nucleotides were counted for each microbial species in each patient, this value was chosen to be stricter than the suggested metagenomic sequencing guideline (75 bp) to ensure accuracy in a clinical setting^13^. Read counts were normalized based on total rWGS coverage. We abstracted electronic health record data of all patients who had rWGS performed at Rady Children’s Institute for Genomic Medicine (RCIGM) between July 2016 and July 2019. In patients for whom charts were available in the electronic medical record, a manual review was performed by a pediatric infectious disease fellow (NR) to determine if the patients had clinical features consistent with CMV infection and/or positive clinical plasma CMV PCR results (DiaSorin Molecular CMV Primer Pair and the LIAISON MDX Thermocycler, Cypress, CA, USA). In false negative patients who received rWGS testing and were positive for CMV by plasma CMV PCR, but were negative by realignment of unaligned rWGS reads, we performed CMV PCR on the sample used for rWGS. We also performed CMV PCR testing on the saved DNA specimens used for rWGS testing on the patients in whom two or more reads of CMV were detected. Each qPCR run was performed with a high titer and low titer external positive control (Exact Diagnostics, Fort Worth, TX) in addition to a negative control. We performed Sanger sequencing PCR reactions as earlier described using previously validated primers on these DNA specimens to exclude the presence of a PCR inhibitor^1^.

### Ethics approval

Peripheral blood was obtained from ill pediatric patients (age 1 day to 18 years) after parental consent and patient assent or consent where possible according to University of California San Diego Institutional Review Board. Research was conducted according to the principles of the Declaration of Helsinki.

###  Consent for publication

 All patients were consented. When possible patient assent was obtained as well.

## Results

From July 2016 through July 2019, 669 patients received rWGS at RCIGM. Of these, 164 were found to have at least one read that did not align to the reference human genome and that completely aligned to a human herpesvirus (HHV) genome: one subject with reads aligning to HHV-4 (Epstein-Barr virus, NC_009334.1), 28 with reads aligning to HHV-5 (CMV, NC_006273.2), 69 with HHV-6 (NC_001664.2 or NC_000898.1), and 66 with HHV-7 (NC_001716.2). The 28 patients in whom we detected two or more reads of HHV-5 (CMV) represented 4.2% of the total population evaluated. Through chart review we identified clinical characteristics suggestive of symptomatic CMV infection in six of these 28 patients (21%) (See Table 1, case summaries are found in the Supplement).

**Table 1:** Symptomatic patients with CMV identified by rWGS pipeline.

Patient ID	CMV reads (#)	Age	Genetic diagnosis	Patient description	Standard care testing for CMV	TCP	Hep	AVT	Hearing test
Patient 1	3	8 d	Dursun syndrome	Profound neutropenia and cerebral infarct	NT	Yes	No	No	Passed
Patient 2	1,654	6 m	None found	Kaposiform hemangio-endothelioma with pneumocystis pneumonia	CMV plasma PCR negative. CMV serology positive. CMV in lungs on histopathology	Yes	Yes	No	NT
Patient 3	2	9 m	None found	Transient pancytopenia, megaloblastic	NT	Yes	No	No	NT
Patient 4	2	4 d	ASLD	Liver failure	NT	Yes	Yes	No	Passed
Patient 5	634	2 m	Ebstein anomaly	Ebstein anomaly status post heart transplant	Positive plasma PCR (1,892 IU/mL)	Yes	Yes	Yes	Passed
Patient 6	8	13 y	GPA	GPA with disseminated mucormycosis infection	Positive qualitative plasma PCR and bronchoalveolar lavage	Yes	Yes	No	NT

*ASLD* arginosuccinate lyase deficiency, *D* days, *M* months, *Y* years, *GPA* granulomatous polyangiitis, *TCP* thrombocytopenia, *Hep.* Hepatitis, *AVT* antiviral treatment, *NT* not tested.

Of these six patients, three had never been evaluated for CMV infection. Only one patient had been started on antiviral treatment. To assess for false-negative results associated with our detection methods, we performed a chart review of all children who received rWGS testing at RCIGM to identify patients found to have CMV infection by standard-of-care testing. Given the difficulty of interpreting serology, we limited our search to patients with positive CMV PCR tests^14^. We identified five patients who were CMV quantitative PCR (qPCR) positive but not identified by rWGS read re-alignment (Table 1 in Supplementary material). Recognizing that CMV testing and rWGS were often done at different times during the patient’s hospital course, we performed CMV qPCR testing on the same DNA sample (dedicated CMV qPCR) that had been used for rWGS. PCR testing detected CMV in only one of five patients tested. In that case, CMV levels were too low (< 227 IU/mL) to be quantifiable, indicating a very low viral load. We additionally performed a dedicated CMV qPCR on the saved DNA specimen used for rWGS for the 28 patients with two counts or more of reads consistent with CMV (Table 2).

**Table 2:** Patients with 2 reads or more reads of CMV detected in rWGS. CMV PCR was performed on the same DNA sample used for rWGS testing.

Unaligned reads from rWGS	PCR	Sample age (months)
2	Not detected	24
2	Not detected	19
2	Not detected	23
2	Not detected	17
2	Not detected	18
2	Not detected	15
2	Not detected	15
2	Not detected	15
2	Not detected	12
2	Not detected	19
2	Not detected	14
2	Not detected	17
2	Not detected	33
2	Detected, but < 227 IU/mL detection limit	14
3	Not detected	25
5	Detected, but < 227 IU/mL detection limit	14
8	Detected, but < 227 IU/mL detection limit	20
9	Detected, but < 227 IU/mL detection limit	19
11	Detected, but < 227 IU/mL detection limit	16
12	795 IU/mL, 2.9 LOG IU/mL	14
19	Not detected	17
31	132 IU/mL, 2.12 LOG/mL	1
36	345 IU/mL, 2.54 LOG IU/mL	14
45	2,417 IU/mL, 3.38 LOG IU/mL	27
69	1513 IU/mL, 3.18 LOG IU/mL	23
85	1,071 IU/mL, 3.03 LOG IU/mL	15
634	14,581 IU/mL, 4.16 LOG IU/mL	20
1654	45,532 IU/mL, 4.66 LOG IU/mL	29

**Figure 1:**
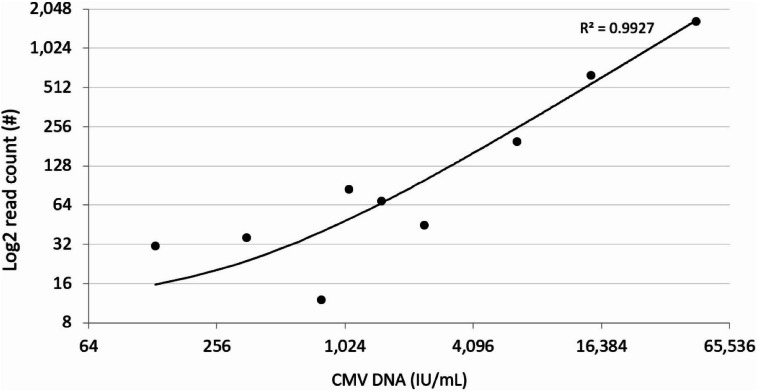
Log_2_ CMV read count from rWGS versus viral load from CMV-quantitative PCR assay for 8 samples where rWGS detected CMV DNA and quantitative PCR could quantify viral load. rWGS read results are highly correlated to infectious unit determination by gold-standard quantitative PCR assay.

To assess for the presence of a PCR inhibitor, we used Sanger PCR reaction on all 28 samples tested. All of the samples underwent testing with two individual validated oligo sets. Each oligo set included one internal amplification control and one negative control. In 13 of the 14 patients with only two unaligned reads consistent with CMV, PCR reaction did not detect CMV. In a patient with three reads of CMV in WGS data and another with 19 reads in the rWGS data, CMV was not detected by PCR. Above a read count of 19, CMV was consistently detected by PCR reaction and quantifiable. The infectious unit count detected by PCR was highly correlated with the read count from rWGS (R^2^ = 0.9942; *p* < 0.0001; see Fig. 1.).

## Discussion

Of 669 subjects who underwent rWGS at RCIGM from July 2016 through July 2019, we identified 28 patients with two or more DNA sequence reads consistent with CMV DNA. We also identified 164 patient genomes with unaligned reads consistent with DNA from any herpesvirus. Chart review identified clinical findings such as hepatitis, seizures, and thrombocytopenia that could be consistent with symptomatic CMV infection in 6 of the 28 in whom the presence of CMV was detected (see Table 1). Moreover, in these 6 case histories, even if the primary diagnosis may overlap with symptomology of CMV infection, CMV may have still have contributed to the symptoms experienced by these patients (and possibly even been the major cause of illness in case number 6). Of these, three patients had not previously been evaluated for CMV, and only one patient had been started on antiviral therapy. Two of the patients (cases 1 and 4) were neonates with possible congenital CMV infection and would have met inclusion criteria for treatment based on Kimberlin et al.^9^ In cases 2, 3, 5, and 6, though it is not possible to distinguish congenitally versus postnatally acquired infection, all of these patients were relatively immunosuppressed as a result of their primary diagnosis or from their treatment regimen.

In 5 cases with a positive clinical qPCR, we did not identify a CMV infection by WGS. In 4 of these cases, a confirmatory qPCR conducted on the plasma obtained for WGS was negative, likely due to a waning viremia. In the remaining case, CMV was detected, but below the detection limit for quantitation. In one case, a substantial HHV7 viremia was detected, and it may be possible that the initial CMV positive was due to cross reactivity with one of the qPCR primers for HHV7. Although qPCR can be highly specific, it is generally tested in the background of subjects with no viremia rather than a different HHV infection^15^. Future studies will need to be conducted to test if WGS can also be more sensitive than qPCR in some cases.

The clinical spectrum of CMV is highly varied and overlaps with many other disease processes. The ability of clinicians to identify active CMV viremia clinically can therefore be challenging^16^. In neonates in particular, even patients with mild disease initially may have significant morbidity, including sensorineural hearing loss^17^. A land mark study by Kimberlin and colleagues in 2015 demonstrated that morbidity from symptomatic congenital CMV infection could be reduced or averted by early initiation of antiviral therapy^9^. Recent studies have explored the utility of hearing screening to identify congenital CMV, though with a sensitivity of just 53%, this strategy will miss many affected patients^8,18,19^. Current diagnostic approaches are inadequate to identify CMV infection in vulnerable patients in a timely manner.

The use of unaligned reads from rWGS testing offers a cost-effective approach to identifying infectious pathogens while simultaneously testing for pathogenic variants in the host genome^20^. Given that most patients undergoing rWGS are critically ill, it is likely that many of these patients have an infectious process superimposed on an underlying medical condition that led to a severe disease state^7,21,22^. CMV, which is associated with significant morbidity in neonates and immunodeficient hosts, is a pathogen that may be implicated in many such cases. Analyzing rWGS for microbial DNA enables the testing laboratory to alert clinicians in a timely manner to the presence of this pathogen while simultaneously evaluating the host genome for other pathogenic conditions. The results of this assay are highly correlated to orthogonal testing with CMV-specific qPCR. Moreover, the additional cost of testing to confirm CMV infection following rWGS is low. Whole genome data may also play a role in understanding the natural history of congenital CMV beyond the identification of apparently symptomatic children. This is especially relevant given the calls for population-based whole genome sequencing of neonates and for population-level screening for CMV, which will identify many more children infected with CMV whose clinical course is uncertain^16^. Additional research using rWGS may help clarify which CMV positive neonates would benefit from potentially toxic antiviral therapy^16^.

This study has several limitations. We conducted our analysis retrospectively, and the patients we identified were therefore not systematically assessed for CMV infection by the treating team during their hospital stay. Furthermore, even in patients who were evaluated for CMV, the rWGS sample was often not obtained at the same time as the evaluation for CMV, which is significant given that CMV viremia may wane over time or resolve altogether^23^. Our chart review was also limited by the wide clinical variation in what may be attributable to CMV infection (hepatitis, cytopenia, etc.), and in most of our patients, their underlying metabolic or genetic disease process was also associated with many of the classic laboratory findings associated with CMV infection. PCR testing on the 28 samples from patients in whom two or more reads of CMV were detected by rWGS was done on DNA extracted from whole blood samples rather than on plasma as is traditionally done when CMV viremia is suspected. However, the common practice of obtaining plasma CMV PCR in the clinical setting is not necessarily due to a limitation in detection of CMV from whole blood^24,25^. Additionally, given the negative qPCR results for the majority of patients with only 2 reads of CMV and the positive correlation between read count and higher PCR quantitation, there is the intriguing possibility that rWGS may be more sensitive than qPCR for CMV, an observation that has been made for other organsims^26^. Further studies are required to prospectively compare and correlate CMV, and potentially other microbe, read count from rWGS with standard care testing, define the specificity and sensitivity of this approach, and ultimately to demonstrate clinical utility.

rWGS may be a rapid, extremely sensitive method to detect some microbial infections. We propose that, in critically ill children under going rWGS, unaligned reads be used to assess the presence of microbial DNA and that this analysis should be conducted regardless of prior clinical diagnosis. This approach has the potential to identify clinically relevant pathogens such as CMV, which may cause significant disease in certain vulnerable patient populations. Moreover, for CMV infections in particular, early initiation of antivirals may positively modify patient outcomes.

## Supplementary information

Supplementary information
