# Supplementary material for: Diagnosis of cytomegalovirus infection from clinical whole genome sequencing
Source: Sci Rep. 2020 Jul 3;10:11020. doi: 10.1038/s41598-020-67656-5 (PMC7335102; doi:10.1038/s41598-020-67656-5)
Supplement: Supplementary file 1 — Supplementary information [file 41598_2020_67656_MOESM1_ESM.docx]

**Supplement**

Diagnosis of Cytomegalovirus Infection from Clinical Whole Genome Sequencing

Nanda Ramchandar, MD^1^, Yan Ding, MD^1^, Lauge Farnaes, MD^1^, David Dimmock, MBBS^1^, Charlotte Hobbs, MD^1^, Stephen F. Kingsmore, MB^1^, Matthew Bainbridge, PhD^1^*

^1^ Rady Children’s Institute for Genomic Medicine, [7910 Frost St Ste 240, San Diego, CA 92123](https://www.bing.com/local?lid=YN873x10630816249054641666&id=YN873x10630816249054641666&q=Rady+Children%27s+Institute&name=Rady+Children%27s+Institute&cp=32.800758361816406%7e-117.1541976928711&ppois=32.800758361816406_-117.1541976928711_Rady+Children%27s+Institute&FORM=SNAPST) (858) 966-4997

*Corresponding author, [MBainbridge@rchsd.org](mailto:MBainbridge@rchsd.org)

|  | **Herpes virus isolated in genome** | **Confirmatory qPCR** |
| --- | --- | --- |
| Patient 7 | 7 reads of HHV 7, 5 reads of HHV6 A | negative |
| Patient 8 | 47 reads of HHV7, 4 reads of HHV4 | negative |
| Patient 9 | no herpesviruses | detected, but below detection limit |
| Patient 10 | no herpesviruses | negative |
| Patient 11 | no herpesviruses | negative |

**Supplement Table 1** Patients with positive CMV qPCR from inpatient stay not detected by rWGS pipeline

**Case summaries**

**Patient 1**

Patient one was a male who received rapid whole genome sequencing (rWGS) on day of life (DOL) eight, and was diagnosed with Dursun syndrome. He was born term via spontaneous vaginal delivery to a 28 year old female at a community hospital. He was cyanotic at birth, requiring significant respiratory support due to a right-sided pneumothorax. He was intubated prior to transport to a level four neonatal intensive care unit (ICU). He developed pulmonary hypertension and was started on sildenafil. He was also found to have profound neutropenia (absolute neutrophil count of 344 cells/uL) requiring granulocyte colony stimulating factor injections. He developed seizures on DOL two and was found to have a left middle cerebral artery infarct that was deemed non-operative. He was thrombocytopenic (100,000 platelets/uL) at the time of genetic testing, but did not have hepatitis. He was not tested for cytomegalovirus (CMV) nor placed on ganciclovir.

**Patient 2**

Patient two was a 6-month-old male at time of rWGS. He had a history of kaposiform hemangioendothelioma and was on long term immunosuppressive therapy (sirolimus and corticosteroids). At 6 months of age, he presented to the emergency department (ED) of a tertiary care hospital with sepsis and was found to have *Pneumocystis* pneumonia. He was initially inconsolable, mottled, and intermittently apneic. He was intubated on admission to the ICU in fluid refractory hypotensive shock, requiring multiple inotropes. He was referred for genetic testing due to possible immunodeficiency. He passed away from overwhelming sepsis and was discovered to have CMV pneumonitis on autopsy. Plasma PCR for CMV at 5 months of age (40 days prior to rWGS) was negative. Serology was positive for both CMV IgG and IgM 2 weeks prior to rWGS. He had both thrombocytopenia (103,000 platelets/uL) and hepatitis (serum aspartate aminotransferase [AST] 1,130 U/L and alanine aminotransferase [ALT] ALT 629 U/L). He was never placed on ganciclovir.

**Patient 3**

Patient three was a 9-month-old female at time of rWGS who presented with pancytopenia of unknown etiology. She was admitted with bronchiolitis and pancytopenia. Her hemoglobin was 5.9 mg/dL on admission with leukopenia (white blood cell count 5,200/uL) and platelets of 29,000/uL. She underwent bone marrow biopsy, which showed hypocellularity that was not consistent with leukemia or aplastic anemia. All cell lines eventually recovered. She was not tested for CMV. She was thrombocytopenic (19,000/uL), but did not have hepatitis. She was not treated for CMV.

**Patient 4**

Patient four was a 4-day-old male at time of rWGS who presented with liver failure and ultimately required liver transplant. His parents, who brought him to the ED on DOL three, reported the baby was feeding poorly. A sepsis evaluation was initiated, and the patient was admitted to the neonatal ICU. He developed altered mental status and was found to have hyperammonemia (870 umol/L) and hepatitis (AST 163 U/L and ALT 55 U/L). Genetic testing revealed arginosuccinate lyase deficiency. CMV testing was not performed. He was thrombocytopenic (93,000/uL). He was never placed on ganciclovir.

**Patient 5**

Patient five was 2 months old at time of rWGS. She presented with prenatally-diagnosed Ebstein anomaly and ultimately received a heart transplant. She was found to be plasma CMV PCR positive prior to transplant (1,892 IU/mL) at 4 months of age. She was both thrombocytopenic (85,000/uL) and had hepatitis (AST 59 U/L and ALT 60 U/L). She was treated with ganciclovir at therapeutic dosing and then transitioned to valganciclovir at prophylaxis dosing.

**Patient 6**

Patient six was a 13-year-old female patient at time of rWGS. She presented to the ED in respiratory distress. rWGS did not identify a genetic cause of disease. She was found to have granulomatosis polyangiitis and disseminated *mucormycosis* infection. She developed acute respiratory distress, requiring intubation. While intubated, she was noted to have frank pulmonary hemorrhage. She required inotropic support for fluid refractory hypotensive shock. She passed away from overwhelming sepsis and multiorgan dysfunction. She had evidence of CMV on bronchoalveolar lavage by next generation sequencing, and positive CMV plasma PCR (qualitative test). She had thrombocytopenia (130,000/uL) and mild hepatitis (AST 149 U/L and ALT 114 U/L). She did not receive antiviral treatment and passed away before the results of a plasma CMV PCR returned. rWGS testing was done three days prior to her death.
